# The inevitable future of peer review: Human and AI integrated peer review system

**DOI:** 10.12669/pjms.41.4.11941

**Published:** 2025-04

**Authors:** Faaiz Ali Shah, Shaukat Ali Jawaid

**Affiliations:** 1 Faaiz Ali Shah, Associate Professor Orthopaedics Lady Reading Hospital, Peshawar, Pakistan; 2 Shaukat Ali Jawaid Chief Editor, Pakistan Journal of Medical Sciences, Karachi, Pakistan

The traditional peer review process was described by Ernest Hart, Editor of British Medical Journal in 1893 as a “laborious and difficult method, involving heavy daily correspondence and constant vigilance to guard against personal eccentricity or prejudice, or-that bugbear of journalism-unjustifiable censure.”^1^ The technological revolution of present day has added further challenges to those mentioned by Ernest Hart more than a century ago. The workload of editors and peer reviewers has tremendously increased in recent times due to unpresented increased submissions and it has been estimated that 2300 Elsevier journals receive 1.2 million manuscripts every year and only 350,000 (30%) are published.^2^ Furthermore the rejected manuscripts consume more than 15 million hours of peer reviewers precious time each year.^3^

In 2024, the *Pakistan Journal of Medical Sciences* initially screened and accepted 2,423 manuscripts for further processing. However, only 555 manuscripts were published after peer review. Most submissions came from China (1,095), Turkey (192), and Saudi Arabia (117). After peer review, the number of accepted and published manuscripts was 193 from China, 26 from Türkiye, and 29 from Saudi Arabia.

The current peer review process is overburdened and strained. Although research output has increased many folds over the years but the size of peer reviewers community remains the same. About 20% of the researchers tackled 94% of all peer reviews resulting in 18.9 million hours (30%) provided by only top 5% researchers out of total 63.4 million hours spent on peer reviews in 2015.^4^As peer review is considered as an unpaid, spare time academic activity subject to the good will of researches, a smaller number of researchers are willing to accept review invitations because of increased workload and peer reviewer’s fatigue. Journal editors often struggle to find expert peer reviewers. The existing pool of peer reviewers is limited and lack inclusive and diverse peer reviewer.

The *Pakistan Journal of Medical Sciences* has consistently worked to recruit and retain quality reviewers by offering various incentives. Our reviewer database now includes over 750 reviewers, with approximately 74% from overseas. Reviewers who submit their own manuscripts receive a waiver on processing fees and discounts on publication charges, a benefit available to both local and international reviewers. Additionally, selected reviewers—particularly newly inducted ones—are offered a token honorarium for reviewing manuscripts. Despite these efforts, finding high-quality reviewers remains a challenge. The *Journal of the College of Physicians & Surgeons Pakistan* has also provided honoraria to its reviewers for several years to address this issue. However, for experienced and committed reviewers, their primary motivation lies in professional recognition, respect, and association with reputable peer-reviewed journals rather than financial incentives.

Different Artificial Intelligence (AI) tools have been developed to help publishers, editors and peer reviewers in different stages of peer review process (Table-I)^5^,^6^ including peer reviewer selection, manuscript screening for quality control and artificially generated text detection. These AI tools have been shown to reduce 73% editorial time for allocating manuscript to a suitable reviewer and 37% additional reviewers’ suggestions initially not considered by editors.^7^ These tools are helpful to uncover and reduce reviewers’ biases.^8^ They are useful in pre review editorial screening for potential research integrity like paper mill submissions, violation of reporting guidelines and image manipulation which are usually difficult to detect by peer reviewer. Because of these benefits AI tools need to be integrated in peer review process by publishers, editors and peer reviewers. This will speed up peer review process without affecting the quality of peer review process.

**Table-I:** Different AI software tools applicable to peer review system.

S. No	Name of AI Tool	Function
1	ZeroGPT	Detection of text generated by Chat GPT
2	SciScore	Assessment of Methods
3	RobotReviewer	Assessment of study designs and bias
4	StatReviewer	Assessment of Statistical analysis.
5	StatCheck	Assessment of consistency of statistical reporting based upon *P* value
6	Dimensions Research Integrity preCheck	Assessment of Transparency and Reproducibility of manuscript
7	Penelope.ai	Assessment of submitted manuscript for journal requirement.
8	Recite	Assessment of reference matching with citations in text.
9	AuthorOne	Assessment of manuscript formatting
10	Clarivate’s Reviewer Locator	Peer reviewer suggestion based upon Web of science and Publons peer reviewer data bases. [Scholar One Submission system]
11	Reviewer Discovery	Automatic peer reviewer suggestion [Editorial Manager System].
12	Elsevier’s EVISE	Identify and recommend suitable peer reviewer based upon Scopus peer reviewer data.
13	Frontiers Coronavirus Reviewer Recommender	Suggests peer reviews for manuscripts focusing on COVID-19 related research.
14	Review Advisor	Assessment of manuscript matching for journal submission.
15	pReview	Automatically generates summary of submitted manuscript and suggestions for improvement.
16	Peer Read	Predicts the acceptance or rejection of submitted manuscript based upon peer reviewer comments.
17	Peer Judge	Assess the strength and limitations of peer review reports [Sentiment Detection]
18	UNSILO	Summarization of key concepts of submitted manuscript

Currently, no professional societies for medical editors endorse the use of AI tools for peer review. The *World Association of Medical Editors (WAME)* states that individuals in scholarly publishing may use AI-based tools for simple word processing tasks (akin to grammar-checking software), idea generation, and substantive research. WAME also advises that editors and peer reviewers should disclose any use of AI tools in manuscript evaluations, reviews, and correspondence. If AI-generated content is included in communications, users should clarify how it was utilized. 9

The *International Committee of Medical Journal Editors (ICMJE)* restricts the use of AI in peer review and requires reviewers to seek permission from journals before employing AI technologies in the review process.^10^ Similarly, the *National Institutes of Health (NIH)* and the *U.S. National Science Foundation (NSF)* prohibit the use of AI tools in peer review and application evaluations. The *Committee on Publication Ethics (COPE)* has also issued guidelines emphasizing transparency and appropriate disclosure regarding AI use 11 However, journal editors must balance AI’s capabilities with the human expertise necessary to uphold the integrity of the peer-review process.

Peer review is a complex process and AI tools cannot replace or substitute human judgement and expertise. A hybrid approach complementing AI tools and human judgement can combat the current and future challenges of peer reviewers. The delicate balance between AI innovation and responsibility can only be ensured with human supervision comprising of transparency, accountability and confidentiality. This human supervision has rightly been termed as “Humanity of Peer Review” by Karan^12^ and is critically important for the success of this transformation.

“AI is love in Chinese” 爱 = Ài.^13^ Use of AI in the peer review is just the begging. We believe what Stephen King has said that the “scariest moment is always just before you start. After that, things can only get better.” Peer review is constantly evolving and the critiques of AI integration will resolve as the system evolves. Human judgement and AI capabilities will converge for the effectiveness of this long peer review journey. The question should not be to allow or not to allow AI in Peer review process but should be how to execute a balanced and responsible integration of AI in Peer review process. Human and AI integrated peer review is the inevitable future of peer review. Publishers, editors and peer reviewers should embrace it rather than avoiding it.**14**To ensure smooth and effective human and AI integrated peer review process, the publishers, editors and governing bodies should demonstrate commitment and revise their publication and peer review policies and guidelines for implementations while preserving the integrity of peer review.

One must not afraid to change for good. In the words of Winston Churchill “To improve is to change; to be perfect is to change often.”
